# Analysing Deception in Witness Memory through Linguistic Styles in Spontaneous Language

**DOI:** 10.3390/brainsci13020317

**Published:** 2023-02-13

**Authors:** Sara Solà-Sales, Chiara Alzetta, Carmen Moret-Tatay, Felice Dell’Orletta

**Affiliations:** 1 Doctoral School, Catholic University of Valencia San Vicente Mártir, San Agustín 3, Esc. A, Entresuelo 1, 46002 Valencia, Spain; 2 ItaliaNLP Lab, CNR-Institute for Computational Linguistics “A. Zampolli”, Via G. Moruzzi 1, 56124 Pisa, Italy; 3 MEB Lab, Faculty of Psychology, Universidad Católica de Valencia San Vicente Mártir, 14600 Valencia, Spain

**Keywords:** simulated deception, natural language processing, content analysis, linguistic cues, witnesses

## Abstract

The act of lying and its detection have raised interest in many fields, from the legal system to our daily lives. Considering that testimonies are commonly based on linguistic parameters, natural language processing, a research field concerned with programming computers to process and analyse natural language texts or speech, is a topic of interest on this front. This study aimed to examine the linguistic styles of simulated deception and true testimonies collected with the aim of studying witness memory. Study participants were asked to act as a witness of a crime by retelling the story they had just read. Cognitive interviewing techniques were used to collect testimony under two conditions: truth and simulated deception. A sample of 48 participants volunteered to participate in the study. Analyses of the linguistic indicators and content were carried out. Specifically, we performed a comparison of testimonies of the same participant by condition to analyse the variation between (i) lexical and (ii) linguistic features and (iii) content and speech characteristics (disfluencies) depending on the narrative condition. Concerning lexical properties, adjectives were the most-varying grammatical category between truthful and deceptive testimonies. Furthermore, in the linguistic analysis, we observed that truthful testimonies were generally longer than deceptive ones in terms of the number of words and sentences and also characterised by more articulated sentence structures, and these differences were also statistically significant. Regarding the analysis of the content, cognitive criteria (details) and admitting lack of memory were more present in truthful statements. By providing an objective measure, these results are of interest in developing NLP tools for assessing the credibility of testimonies in forensics.

## 1. Introduction

Memory is described as a reconstructive process from reality [1,2], which implies a subjective interpretation of someone’s speech or writing [3]. Not surprisingly, the assessment of eyewitness testimonies can be considered a difficult task. Moreover, when an individual intentionally lies, two types of deception might appear: (i) creating new information (primary deception); (ii) concealing the difference between the statement and the intention to narrate it credibly (secondary deception) [4]. Focusing on intentional deception is of interest to different fields through credibility, from forensics to linguistics. However, linguistics does not seem to have delved into the nature of this phenomenon since the last decade [5].

Artificial intelligence has become a promising tool in this field. More precisely, Natural Language Processing (NLP), which is based on the premise that language is a cognitive process that underlies executive functions and memory, among others, could be of interest on this front. In this way, the current study was framed in the contributions that both psychology and linguistics can offer to witness testimony.

Witness testimony can be one of the most compelling types of evidence in a trial due to its underlying credibility, which is also the most subjective factor to be examined in a process. Several attempts have tried to assess credibility from a more objective way, such as psycho-physiological indicators (galvanisation of the skin, heart rate, sweating, brain changes, among others), to other subjective variables such as non-verbal cues (e.g., vocal and facial features and movements) and content criteria [6,7]. Vocal cues for the detection of deception have been studied extensively, highlighting the presence of interjections, speech errors, and speech rate in lies requiring a higher cognitive effort [8].

To examine credibility, Criteria Based Content Analysis (CBCA) [9] is one of the most accepted techniques. This checklist takes part in the Statement Validity Assessment protocol, which stipulates that the memory of a self-experienced event differs in content and quality from a non-experienced one. However, there is a lack of consensus on the weighting of criteria, which increases sensitivity according to the discriminatory power [10]. The amount of detail and contextual information has been recognised throughout the literature as a predictor of deception by relating it to strategic avoidance of verifiable information [11,12,13]. The revised CBCA technique [14] classifies the criteria into cognitive and motivational. The first group includes episodic autobiographical memory (spatial and temporal details, reproduction of conversations, emotions and feelings, among others) and script-deviant information (superfluous and unusual details, unexpected complications, related external associations, etc.). Motivational criteria refer to the witness’s self-presentation efforts and the manner in which the witness presents the statement. Examples of motivational criteria are: making spontaneous corrections, admitting forgetfulness, or expressing uncertainty. The authors claim that the truth-teller takes advantage of autobiographical memory, while the liar tends to focus on appearing credible. More specifically, Maier et al. [15] investigated the relationship between the strategies employed by liars and the occurrence of criteria. They concluded that, when lying, the strategy of including episodic details is valued as positive. However, it is to be hoped that script-deviant and motivation information will be avoided, as liars find it negative to include them in their testimony. Therefore, the presence of episodic autobiographical details would not directly imply truthfulness.

## 2. Related Work

Contributions are limited if we focus on deception detection in Spanish via NLP in the forensics–psychological domain. However, Vogler and Pearl [13] investigated deception content in three different domains, concluding that linguistic features are more generalisable across domains and that specific details reflect the psychological process underlying the creation of truthful or deceptive content.

Automated analysis of the content of a text allows the extraction of linguistic features. General Inquirer [16] was one of the first systems developed for this purpose. This system explores the frequency of words according to their lexical category using sentences as the unit of analysis. Another noteworthy system is CohMetrix [17], which, in addition to word frequency, takes into account the cohesion of words, analysing the meaning and context in which they appear. Finally, LIWC [18] is a word-centred instrument that allows the study of language at the emotional, cognitive, and structural levels. It was used in one of the first studies [19] that applied deception detection through NLP to forensic psychological practice, in particular in its fifth experiment, where a fictitious crime paradigm was carried out.

On the other hand, Zhou’s research group [20] developed Linguistics-Based Cues (LBCs) through a study in which they analysed emails in which the communicator had been truthful or untruthful. To do so, they synthesised features provided by classical content analysis tools, such as CBCA or RM, and linked them to NLP levels of analysis. They found discriminant criteria between testimonies, although many were inconsistent with previous research. Therefore, they stressed the importance of taking into account context (textual versus face-to-face) and deception planning time, related to cognitive load and anxiety.

The DePaulo team’s research [21] is credited with having studied 158 indicators of deception in English-language reports made by adults, drawn from 120 independent samples. This meta-analysis found that liars were less communicative (talking time and details) and convincing, including fewer imperfections (spontaneous corrections and admitting lack of memory) and unusual content and more complaints and negative statements. In addition, it was concluded that deception cues were more pronounced when there was a motivation for success, especially if it was identity (as opposed to material motivation).

In the same vein, Hauch and colleagues [22] conducted a meta-analysis of 79 indicators of deception studied in 44 research studies whose sample was mainly adult speakers of different languages, mostly English. In addition, texts were collected from real cases, crime simulations, attitudes, and others, through different types of communication where the motivation varied from null to high. These indicators were grouped into six research questions explored using LIWC. Broadly speaking, they showed that lying entails higher cognitive load, negative emotions, more distance from the event, and fewer sensory–perceptual and cognitive process references. However, no greater insecurity was found in lying.

In relation to romance languages, Fornaciari and Poesio’s research [23] is remarkable for having been carried out in a real-life context. The corpus used was collected from court hearings in Italy of persons accused mainly of slander or false testimony. Linguistic feature extraction was carried out with LIWC software, and their results showed a higher presence of “yes”, spatio-temporal information, and positive feelings in truthful testimonies. In contrast, the false testimonies were characterised by a higher frequency of the word “no”, negative feelings, expressions of lack of memory, and first-person pronouns.

Finally, in the Spanish language, the presentation of Veripol [24] is noteworthy. This is a model that combines NLP and Machine Learning (ML), developed together with the National Police, for the detection of false reports of theft, obtaining a hit rate of over 91%. In addition, this research illustrates the differentiating characteristics between true and false texts. Morphosyntactically, they found that false reports are characterised by reflexive missing and shorter sentences, negations, common nouns, and common and non-reflexive verbs. This indicates that, in untruths, they tend to be more impersonal in their narration, creating a distance from the facts. In terms of detail, truthful texts tend to be longer and richer in detail.

In Spanish also, reference can be made to Almela’s study [5]. This research explored, through LIWC, the linguistic keys of written opinion texts on homosexual adoption, bullfighting, and feelings about a good friend. The main results described truthful texts by a higher presence of first-person verbs in the past and future tense, sensory–perceptual words, insight words (e.g., think), tentative words (e.g., maybe), and exclusive words (e.g., but). On the other hand, the false texts presented shorter 2nd- and 3rd-person responses and words related to negative emotions.

All in all, there seems to be agreement on some aspects related to lying, such as cognitive complexity. On the other hand, some debate is found in some indicators of lying such as insight words or expressions of insecurity. For this reason, it is essential to take into account moderating variables. Therefore, it is important to emphasise that this study was conducted in Spanish, in a face-to-face interview, with no planning time and low motivation to lie, bearing in mind that the success of this does not have major consequences for the participants.

In sum, this study aimed to describe the differential linguistic styles between truth and intentional deception. For this purpose, the testimonies obtained from a free recall task were studied through three dimensions: lexical, linguistic, and content and speech. In this way, differences between true and simulated deception testimonies were expected. More precisely, it was hypothesised that deceptive testimonies depict higher variations in terms of lexical, linguistic features, and content than true testimonies. Nevertheless, it was also hypothesised that there are no speech disfluencies between truth and deceptive testimonies, as, in deception, the aim is to produce a discourse as close to the real one as possible.

## 3. Method

### 3.1. Participants and Ethics

A call for interest providing general information about the study was disseminated through social networks between March and May 2022 so that interested candidates could contact the researchers. Since the study was conducted in Spanish, being a Spanish native speaker was an essential requirement for participants. Other inclusion criteria concerned age, i.e., between 18 and 65 years of age, and not suffering comprehension difficulties.

Eventually, researchers recruited 48 volunteer participants who satisfied the requirements. The sample was balanced with respect to the gender of participants, and it involved people aged between 18 and 63 (AVG = 38.87, SD = ±12.70). The sample showed a heterogeneous education level: primary level (25%), secondary level (31.25%), higher education (20.83%), and post-university education (22.91%). Furthermore, in the *Comunidad Valenciana*, where the study was conducted, there are two official languages. Thus, all participants spoke Spanish as L1 and Valencian as L2.

The study was carried out in accordance with the Declaration of Helsinki, and it had the approval of the committee UCV/2021-2022/060.

### 3.2. Materials

The study relied on two crime stories adapted from [25], which were based on Helm’s investigation [26]. The two stories describe a robbery and consist of about 140 words, complemented by 4 pictures showing the following characters: a male robber, a female victim, and two men intervening to help the victim. The stories also feature two friends witnessing the crime. The two stories diverge with respect to the characters’ physicality, the spatio-temporal context, and the manner in which the robbery was carried out. In order to make the stories more relatable for the participants, we translated them into Spanish and set the events in the city of Valencia.

The text of both stories and the corresponding pictures are fully reported in Appendix A.

### 3.3. Procedure

The study of witness memory was carried out in individual sessions articulated in two main phases. During the first phase, the researcher provided general information about the study (research team, duration, ethics committee, the possibility of withdrawal at any time, etc.) and data protection to the participant.

The second phase consisted of a 20–30 min recorded interview. The recommendations proposed by the cognitive interview were followed [27]: a calm atmosphere was promoted by pointing out that there were no right or wrong answers. The first prompt was to read one of the two stories carefully taking as much time as needed. In order to achieve greater ecological validity, a cognitive stress condition was added after reading the story, which consisted of performing a backward counting task, 3 by 3, starting from 100. Then, the participant was asked to mentally reconstruct the story, placing himself/herself in the event and thinking about the emotional and contextual elements of the narration, which is a known practice to facilitate recall [28]. To do this, they could take as much time as they needed and even closed their eyes. Once ready, the participant performed a series of narration tasks pretending to be one of the two witnesses of the crime testifying in a trial, thus including all details regarded as relevant. The first task—referred to as *truth*—consisted of retelling the story just read as-it-is. In the second task, the participant was instructed to lie about the identity of the thief, accusing a different character of the story. This second condition is referred to as *simulated deception*. For both tasks, the participant was asked to narrate the events in linear and reverse order to increase the likelihood of remembering details and reduce the influence of expectations and schema [7,27]. The procedure was repeated for the second story before concluding the interview. Note that the order in which the stories were read was counterbalanced into two groups to avoid bias. Overall, we collected 384 testimonies: 192 for each of the two narrative conditions.

### 3.4. Corpus Preparation and Indicators of Variation

Participants’ retellings collected during the interviews were manually transcribed to obtain the corpus of testimonies, internally divided into two sub-corpora of truthful and deceptive retellings. For the purposes of the transcription, we added some “natural punctuations” [29] (i.e., periods and commas) according to speech pauses and intonations to identify major utterance boundaries, roughly corresponding to sentences. As a further step, we defined a set of indicators, detailed below, that concern stylometric and speech properties that may vary in the testimonies based on the narrative condition.

#### 3.4.1. Stylometric Properties

The variation across testimonies based on stylometric analysis investigates the differences between retellings with respect to surface-related and linguistic features [30]. Stylometry relies on computational methods for automatically extracting low-level verbal cues from corpora in order to acquire the linguistic profile of a text. Accordingly, we relied on surface-related features to capture the lexical variation between truth and simulated deception testimonies, while linguistic features allowed us to assess whether deeper syntactic phenomena occurring in participants’ productions varied based on the narrative condition.

To perform the lexical analysis, we acquired frequency lists of words (unigrams) from the full corpus of testimonies and from the two sub-corpora of truthful and deceptive retellings. To reduce the data sparsity, frequency lists were constructed based on words’ base forms, namely their lemma (e.g., “ver” (see) for “vimos” (“we saw”), “hombre” (man) for “hombres” (men), etc.) and grouped by grammatical category (Parts-Of-Speech (POSs)).

Linguistic features, on the other hand, capture the deeper linguistic variation driven by the narrative condition. The approach to studying such variation was inspired by research on *linguistic profiling*, a methodology in which “the occurrences in a text are counted of a large number of linguistic features, either individual items or combinations of items. These counts are then normalised [...]” in order to detect and quantify differences and similarities across texts [31]. Although it was originally developed for authorship recognition or verification purposes, this methodology proved to be effective in multiple scenarios, for example to study variations related to textual genre and register [32] or to the social dimension of language [33].

In this study, we relied on the linguistic profiling methodology described in [34] and implemented in Profiling-UD (tool available at http://www.italianlp.it/demo/profiling-UD/, accessed on 6 February 2023), the first web-based tool conceived of to linguistically profile multilingual texts by relying on the Universal Dependency (UD) formalism [35], a de facto standard dependency-based schema for morpho-syntactic annotation on corpora. This tool computes about 150 features representative of the linguistic structure of a text and derived from raw, morpho-syntactic, and syntactic levels of annotation. In this study, we relied on the 123 most-frequent features occurring in the corpus in order to prevent data sparsity issues.

As can be seen in Table 1, the linguistic features acquired for this study were grouped into 8 main types of linguistic phenomena. They ranged from quite simple aspects related to raw text properties (i.e., sentence and word length), to the distribution of UD Parts-Of-Speech and of inflectional properties specific in particular to verbal predicates (i.e., mood, tense, person). More complex features are related to the global and local syntactic structure of a document, such as the verbal predicate structure, e.g., in terms of the number of dependants of verbal heads, and the order of subjects and objects with respect to their verbal head. We also considered a group of features capturing the use of subordination in terms of the distribution of subordinate clauses, of their internal structure, and the relative order with respect to the main clause.

We chose to rely on these linguistic characteristics since they have been shown to be highly predictive when leveraged by stylometry studies, a dominant approach for studying verbal cues in deception identification (see, e.g., [19,23,36,37]).

#### 3.4.2. Content and Speech Disfluency Properties

Next to linguistic properties, properties related to content and speech were also investigated as indicators of variation between narrative conditions in testimonies [38]. For content properties, we relied on the classification provided in [14], which, as can be read in Section 1, distinguishes between *cognitive* and *motivational* criteria. The former refers to episodic autobiographical memories and script-deviant information (i.e., irrelevant and superfluous details), while the latter captures efforts of positive strategic self-presentation (i.e., attempts to appear credible, such as admitting lack of memory). Speech disfluency properties, on the other hand, concern typical phenomena of spoken language, such as false starts and repetitions. Vocal cues for deception detection have been studied extensively and have been proven effective for identifying untruthful statements [39,40].

For the purposes of this study, a researcher with experience in the analysis of witness testimonies manually annotated content properties and speech disfluency properties on the “raw” transcriptions. To this aim, the annotator relied on a set of “xml-style” labels inspired by those used in [41]. Their description and examples of their use are reported in Table 2.

## 4. Results

In this section, we report the results of a set of quantitative and qualitative analyses aimed at comparing truthful and deceptive testimonies with respect to the stylometric, content, and speech indicators described above (cf. Section 3.4). In particular, we performed a qualitative comparison of the results of frequency distribution analyses carried out on the two sets of retellings, and we accompany them with inferential statistical analyses as described below. Note that we relied on non-parametric statistical tests since the distribution of the variables under study did not satisfy the normal distribution assumption required by parametric tests.

The study investigated two complementary levels of analysis. On the one hand, we compared the two sub-corpora of retellings to identify their main differences in terms of stylometric (namely, lexical, and morpho-syntactical) and content properties. On the other hand, a participant-level analysis allowed us to assess whether the testimonies of the same participant varied with respect to the monitored indicators depending on the narrative condition. Such a more fine-grained analysis abstracts away from one’s personal linguistic style, which tends to be fairly fixed [42], and accounts for the narrative condition as the only variable at play.

Note that we excluded punctuation from these analyses since it resulted from a manual addition on the part of the experimenter in the transcription phase.

### 4.1. Surface-Related Features: Lexical Variations

Our first analysis was devoted to assessing whether the use of the lexicon varies in the sub-corpora of Truthful (T) and Deceptive (SD) testimonies. To perform such an analysis, we relied on the frequency lists of lemmas acquired from T and SD as described in Section 3.4.1. As can be noted from Table 3, which reports the results of the analysis performed on frequency lists, unigrams grouped by POSs were split into *closed-class* and *open-class words*. The former refers to the category of function words (e.g., pronouns, determiners, conjunctions, and prepositions), which are the most-commonly used words in language playing a functional role in the discourse [43,44]. They are distinct from open-class words, which comprise content words that contribute to the meaning of the sentence in which they occur. This class includes nouns, lexical verbs, adjectives, adverbs, and proper nouns. Words that do not belong to the previous classes, such as symbols and interjections, fall in the “Other” group, while “Total” indicates the overall amount of tokens in the respective corpus.

By looking at the frequency distributions reported in Table 3, we noticed that the amount of tokens is higher in T than in SD for all POSs and overall. This resulted in a larger sub-corpus (in terms of tokens) of truthful testimonies, and most importantly, it suggests that participants tend to produce longer narratives when telling the truth. However, if we focus on the relative frequency of the POSs in each sub-corpus, we noticed that these are quite similar in T and SD. In other words, although they may vary in quantity, the proportion of each POS with respect to the other word classes remained constant regardless of the narrative condition. Adjectives represent the only notable exception: both their absolute and relative frequencies were higher in the SD sub-corpus. As observed in past research (see, e.g., [13,45,46]), also in our corpus, the distribution of adjectives was skewed in favour of deceptive testimonies. Pronouns are also typically monitored in deceptive texts, as it was found that witnesses tend to create more distance between them and the events when lying, thus using less personal pronouns [19,22]. In line with previous results, we observed a slightly higher frequency of pronouns in T.

To deepen our analysis, we investigated whether the similarity between the word frequency distributions discussed above also corresponded to a deeper similarity in the use of the lexicon. To this aim, we relied on two metrics, both reported in Table 3, which offer complementary perspectives on the degree of lexical similarity between the two sub-corpora of truthful and deceptive retellings. *Lexical overlap* indicates the rates at which the same words are used in truthful and deceptive testimonies regardless of their distribution. To this aim, it was computed—for each POS and overall—as the ratio of the lemmas appearing at least once in both T and SD and the total amount of distinct lemmas in the full corpus. *Spearman’s rank correlation coefficient*, on the other hand, allows quantifying whether words in common between T and SD also have similar relevance, measured in terms of their frequency, in the two sub-corpora. Accordingly, the Spearman correlation was run for each POS on the frequency rankings of lemmas shared by truthful and deceptive retellings, ordered by decreasing frequency of occurrence in their respective corpus (e.g., we computed the correlation score between the frequency ranking of adjectives occurring in T and in SD). This metric allowed us to verify whether words having the same POS occurred with a similar relative frequency in both sub-corpora. If so, we could claim that participants refer to the robbery using a similar lexicon.

Overall, the lexical overlap between truthful and deceptive retellings was around 50%. As can be noted, the overlap was generally higher for closed- than open-class words, with conjunctions even showing a perfect overlap (100%), which indicates that the two sub-corpora of testimonies use the exact same set of words for this POS. This fact is not particularly surprising: the closed class consists of a quite fixed set of elements with highly grammaticalised roles; thus, they may not be used as consciously as content words [44]. The strong Spearman correlation coefficients (>0.9) observed for almost all closed-class POSs indicated also a similar frequency of occurrence in the two sub-corpora. Notably, and corroborating what was said above, pronouns showed the lowest correlation for this class (0.867), indicating a slightly different frequency of occurrence of shared words for this POS in T and SD.

Open-class words, on the other hand, showed lower correlations than closed classes, especially in the case of nominal, i.e., adjectives (0.667), and verbal modifiers, i.e., adverbs (0.797). As a further remark, it should be noted that these are the POSs showing a lower and higher lexical overlap, respectively. In the case of adjectives, this result indicates that only a few words were shared by the sub-corpora, and their rankings based on frequency were also quite different. A similar difference emerged from the correlation score obtained for adverbs, but the high lexical overlap indicated that adverbs used in truthful testimonies generally occurred also in deceptive ones. This result is even more interesting if compared to nouns, verbs, and proper nouns, which instead showed relatively high lexical overlap and correlation. Indeed, assuming that nouns and verbs are used to convey information about entities and events (in this study: the robbery, the thief, the robbed, and the witnesses), their higher correlation with respect to modifiers seems to suggest that, when lying, participants preferred to alter the qualitative properties of actions and entities rather than the core facts of the stories. It could be argued that this is a consequence of the instructions given to the participants. Indeed, in order to comply with the instructions, nouns and verbs must somehow remain the same in both narrative conditions in order to refer to the robbery and the people involved in the story, while the deception can concern qualitative elements of facts and people, or even participants’ perception of the events as witnesses, linguistically expressed by means of nominal and verbal modifiers.

To offer further evidence for this peculiar fact, consider the word lists in Table 4, which reports the 10 most-frequent words for four open-class POSs in T and SD. When we looked at the lists of nouns and verbs, we noticed that they were in fact quite similar. However, the noun “pelo” (hair) is among the most-frequent nouns in SD, but not in T, suggesting that the physical appearance of the characters in the story was more thoroughly described in deceptive testimonies. This intuition seems confirmed by the lists of most-frequent adjectives: while in T, we found highly frequent words referring to the day of the event (i.e., “caluroso” (hot) in 2nd position in T and 7th in SD; “fresco” (cool) in 10th position in T and not appearing in SD), the most-frequent adjectives in SD were mainly used to describe qualitative physical properties, in particular the colour of the hair and skin of the thief. Interestingly, “negro” (black) and “blanco” (white) were the most-frequent adjectives in T and SD, respectively, indicating that the appearance of the thief in the two sub-corpora was diametrically opposed. For the interpretation of the results, it is important to note that the ethnicity of the characters varied across the stories (see the pictures in Appendix A) and that, for the simulated deception condition, participants were asked to focus the lie on the identification of the thief. Taking this into account, the results indicate that, when faced with the truth condition, the skin colour of the robber was more likely to be emphasised when the robber was African-descended than when the robber was Caucasian. However, when participants were asked to lie with regard to the identity of the robber, there was a perceived tendency to change the ethnicity of the African-descended thief rather than other physical characteristics. This can be deduced from the higher frequency of the adjective “blanco” (white) in the simulated deception condition. In contrast, lying with regard to the Caucasian thief tended to change other physical traits beyond ethnicity. Furthermore, to investigate this fact in more depth, we analysed the set of uncommon adjectives (i.e., occurring either in T or SD): what we noticed is that the thief was usually described as Latin-American in truthful testimonies, while he was more frequently described as “gitano” (gypsy) or “islámico” (Islamic) in deceptive retellings, and the other physical properties mentioned tended to reflect stereotypes associated with these ethnic groups (e.g., “moreno” (brown hair), “violento” (violent), “drogado” (addicted)).

As a final remark on the surface-related properties of the testimonies, it is worth mentioning that the lists of most-frequent adverbs suggest that the high lexical overlap observed for this POS was possibly caused by the frequent use in both sub-corpora of discourse markers (e.g., “entonces” (then), “también” (also), “ya” (now)) and adverbs of time and space (e.g., “detrás” (behind), “antes” (before), “después” (after)). While the latter were used to describe the events in the story, discourse markers do not have a precise meaning, but are quite frequent in speech. The correlation score observed for adverbs (0.797) suggests that the amount of information provided by the witnesses to locate the events in time and space might be quite different in the two sub-corpora. This aspect will be explored in more depth in the content analysis (cf. Section 4.3).

### 4.2. Linguistic Style of Testimonies

The analysis reported in this section is aimed at assessing whether the narrative conditions affect the linguistic style of testimonies. To this aim, we relied on a set of linguistic features acquired from T and SD, exploiting the methodology described in Section 3.4.1. The truthful and deceptive testimonies produced by the same participant were paired in order to perform a participant-level comparison of the extent of variation of the linguistic features depending on the narrative condition. This was carried out based on a two-step analysis. First, we computed the Wilcoxon signed-rank test between paired testimonies to explore which linguistic properties varied significantly between T and SD. Due to space constraints, we report the results of the Wilcoxon singed-rank test in Appendix B.1. Specifically, we report in the table the W score, the *p*-value, and the effect size score *r* [47,48] of the test. Then, for those features showing a significant variance between sub-corpora, we checked (i) their mean values in T and SD and (ii) the impact of the narrative condition on the linguistic productions of participants. The latter analysis was carried out by monitoring the variation between the values of features in the T and SD testimonies of the same participant and specifically checking whether these values increased or decreased depending on the condition. The results of the analysis performed on significantly varying features are reported in Table 5. To check the results obtained on the full set of features, refer to Table A2 in Appendix B.2.

Based on our analysis, we found a selection of around 30% of features that showed a significant variation of their values in the testimonies due to the narrative conditions. These features mostly concerned *Raw Text* properties, the distributions of Parts-Of-Speech (*POS*), and dependency relations (*SyntacticDep*), features regarding global and local properties of the parsed dependency Tree (*TreeStructure*), and the use of Subordination (*Subord*). Lower discriminative power was assigned to the Inflectional morphology of Verbs (*VerbInflection*), while the Verbal Predicate structure (*VerbPredicate*), i.e., the number of dependents of a verbal head, and the linear Order of elements in the sentence (*Order*) turned out to not vary significantly between the two sub-corpora.

This linguistic analysis complemented the study on the lexicon discussed in Section 4.1 in multiple ways. First of all, it confirmed the different distributions observed for certain grammatical categories, such as adjectives and pronouns, both with respect to their *POSs* and the dependency relations linking them to their syntactic head (see the features in the *SyntacticDep* group). Additionally, it allowed investigating in more depth the surface properties of the testimonies, as well as deeper linguistic structures. Concerning the former, consider for instance the length of testimonies. The greater length of truthful narratives, observed above with respect to the overall amount of tokens in the sub-corpora, was also reflected at the level of individual testimonies. In particular, significantly varying features of the *Raw Text* group revealed that testimonies in T showed a higher number of sentences and tokens. If we look at this result in light of the variation analysis, we can also see that slightly more than half of the participants produced longer retellings in the truthful narrative condition, but they used a higher number of tokens in 75% of cases. This might suggest that, even when the retellings had an equal number of sentences, these tended to be longer in the truthful testimonies. However, if we look at the average number of tokens in sentences (feature *tok_per_sent*, cf. Table 5), we noticed that the average sentence length was equal to about 20 tokens in both sub-corpora, and rightfully considered as non-discriminative for deception. This indicates that the length of sentences was consistent across conditions, and the tendency to produce longer truthful testimonies was proper for a subset of participants, who simply produced a higher number of sentences.

Such a difference was reflected also in the local and global structure of the parsed dependency trees representing the syntactic structure of sentences. Features falling in the *TreeStructure* and *Subordinate* groups showed that, although all sentences had a similar length, they were characterised by a different deeper syntactic structure depending on the narrative condition. Testimonies of the T sub-corpus, for instance, showed higher use of subordination (feature *subordinate_proposition_dist*) and of embedded nominal modifiers (feature *n_prepositional_chains*), as shown in the following sentence excerpt acquired from the T corpus: *“tenía un poco de cara de mala hostia”* (trad. “he had a bit of a grim face”), where the underlined noun *“hostia”* is syntactically dependent of *“cara”*, which in turn has *“poco”* as its syntactic head. Deceptive testimonies, on the other hand, showed on average longer clauses (feature *avg_token_per_clause*). These elements seem to point to a higher complexity of truthful testimonies, confirming what was stated by [21]: creating and managing misinformation are more cognitively demanding than telling the plain truth; thus, shorter and simpler testimonies are produced when lying.

A further result emerging from this analysis concerns the inflectional morphology of verbs (*VerbInflection* group). As can be noted from Table 5, the presence of verbs in the present tense seems to have a discriminative role for truthful testimonies. Investigating this fact in more depth, we noticed that this was due to a higher presence of linguistic edges, i.e., words or phrases used to express ambiguity, probability, caution, or indecisiveness. These were primarily expressed by means of some verbs: *creer* (to believe), *recordar* (to remember), *saber* (to know). This is in contrast with what was observed in previous studies, such as [49], which found a higher presence of these expressions in deceptive texts. It is essential to analyse the phenomenon behind the emergence of these verbs related to cognitive processes, as detailed in the discussion.

### 4.3. Content and Speech Disfluencies Analysis

The analysis of content and speech characteristics was carried out on the basis of the tags manually annotated on the transcripts, using the procedure described in Section 3.4.2. A descriptive comparison was then made for each property. As for the participant-level analysis carried out on linguistic features, we paired the retellings of participants based on the narrative condition in order to monitor whether a property is more, equally, or less present in truthful than deceptive testimonies.

Regarding the analysis of the content, which concerns *cognitive* and *motivational* criteria, the results displayed in Table 6 show that the former were more highly present in T. Motivational criteria, on the other hand, were mostly equally present in the sub-corpora (see column “Variation”). However, if we look at this result in light of their absolute frequency, which indicated a higher number of motivational tags in T, we could imagine that their distribution was skewed in the sub-corpora. Consider, for instance, the tag capturing cases of lack of memory: the variation analysis showed that, in 75% of cases, the participant was not affected by the narrative condition when recalling the story, which indicated that retellings showed the exact same number of <lm> tags. Looking at this result in more depth, we noticed that the tag was not used in almost all of these cases (97.23%), meaning that the participant did not experience any lack of memory when retelling the stories. By looking again at the tag distribution over the whole corpus, we can conclude that the lack of memory was experienced by only some participants (56.25% of subjects taking part in the study) who tended to express it in most of their retellings, but with a higher frequency in the truth condition.

With respect to speech disfluencies, they did not seem to vary in the truthful and deceptive testimonies as their distribution was quite balanced in the two sub-corpora. However, it should be noted that some tags were heavily more present than others. This was the case of *repetition* and, most notably, *hesitation*. The latter captured cases of speech fillers, namely sounds filling a pause in utterances (e.g., “uhm”, “eeh”), which indeed quite common occur in narrative tasks.

**Figure 1:**
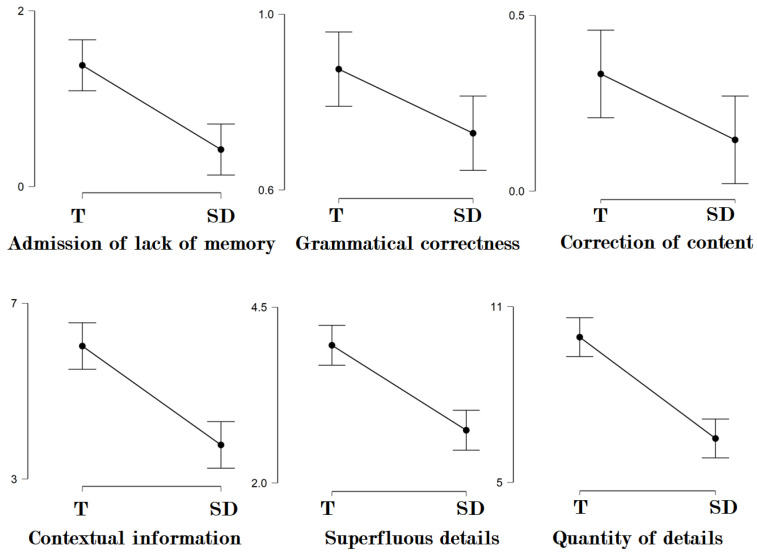
Error bar charts for linguistic style conditions under analysis with statistically significant differences across True (T) and Simulated Deception (SD) testimonies.

Figure 1 depicts error bar charts for conditions with statistically significant differences under the Wilcoxon signed-rank test. In relation to the cognitive criteria, all analyses reached the level of statistical significance. More precisely, differences between T (Mdn = 6) and SD (Mdn = 3) in contextual information reached the significance level (W = 967, *p* < 0.001 ), as well as differences between T (Mdn = 3) and SD (Mdn = 2) in superfluous details (W = 634, *p* < 0.001) and differences between T (Mdn = 9) and SD (Mdn = 5) in the quantity of details (W = 1102, *p* < 0.001). The same pattern occurred for motivational criteria, where the Wilcoxon signed-rank test showed that differences between T (Mdn = 1) and SD (Mdn = 0) in admission of lack of memory reached the significance level (W = 286, *p* < 0.001), as well as differences between T (Mdn = 0) and SD (Mdn = 0) in spontaneous correction of content (W = 104, *p* < 0.05). Lastly, and in relation to speech disfluencies, grammatical correctness also depicted statistically significant differences between T (Mdn = 1) and SD (Mdn = 0), W = 21, *p* < 0.05. Other analyses did not reach the level of statistical significance in this condition.

## 5. Discussion and Conclusions

In this work, we introduced a study based on a free recall task aimed at collecting 384 truthful and deceptive retellings of two stories describing a robbery. To this aim, we recruited a balanced sample of 48 volunteering participants acting as witnesses to the robbery and pretending to be testifying in a trial. Our main goal was to assess whether there was a difference in terms of linguistic style in the testimonies based on the narrative condition in which these were produced. To this aim, we created a novel corpus of truth and deceptive testimonies in Spanish, and we proposed a methodology that integrates the psychological and NLP perspective for studying the impact of deception in the linguistic style of the collected testimonies. In particular, we explored the differences between truthful and deceptive retellings by monitoring multiple stylometric dimensions, concerning the lexical, morpho-syntactic, and content and speech properties of the testimonies. Although relatively small, the corpus of retellings allowed us to conduct investigations on the stylistic differences driven by the truth and simulated deception narrative condition, which we now discuss in light of the literature.

The results obtained based on our analysis highlighted stylometric differences between the two groups of retellings (i.e., truthful and deceptive) and showed how lexical, linguistic, and content and discourse features interact with each other in providing the expressive style of each condition. One of the main results emerging from our analysis concerns the use of the lexicon, which did not seem to be strongly affected by the narrative conditions, as discussed in Section 4.1. Nevertheless, we attested to a slight difference in the use of nominal and verbal modifiers, as well as pronouns, in the two sub-corpora.

In accordance with the literature [5,19,22], such variation concerning nominal modifiers seemed to indicate a tendency to focus lies on qualitative elements rather than on the central facts of the stories, possibly creating a personal distance from the facts. In particular, it is important to highlight two aspects of this analysis. The greater presence of adjectives (hot, cool, contrary, etc.) and adverbs (behind, before, after, etc.) in the sub-set of truthful testimonies indicated a greater presence of descriptive content.

On the other hand, when participants must lie in the identification of the thief, a racial bias seemed to emerge. In particular, there was a tendency to emphasise the skin colour of the African-descended thief, as opposed to the Caucasian. Furthermore, this physical trait prevailed over other characteristics that might describe the African-descended thief. At this point, it is also important to consider how stereotypes can influence the memory of witnesses [50,51]. This can be explained by the cross-race effect, that is there is a tendency to perceive the exogroup under general characteristics, losing the sensitivity to detect individual attributes [52,53].

Regarding linguistic analysis, the main differences that emerged concerned the length of the text, the distributions of parts-of-speech and syntactic relations, the structure of the parsed dependency tree, and the use of subordination. Since the linguistic properties acquired using Profiling-UD showed a high and significant correlation with the perceived complexity of texts [34], we could claim that the true condition results in retellings with higher syntactic complexity than simulated deception ones. These findings are in line with other research in both Spanish [5,24] and in other languages [19,22]. There seems to be a consensus in concluding that lying entails a greater cognitive load [40,54], including, neurologically, a greater activation of the frontal lobe [55]. In particular, to lie properly, it is necessary to be able to multi-task (suppressing the truth, creating new information, appearing credible, etc.) [56]. Multiple resource theory [57] explains how task performance can be affected by several factors: task demand (difficulty), resource overlap (if two tasks require the same resources, interference may occur), and resource allocation (executive control prioritises tasks, with more errors occurring in tasks that are in a secondary position).

In terms of the cognitive criteria for content, our results concluded that truthful testimonies tend to include a greater amount of details, including contextual and superfluous ones. As we have seen, this conclusion is supported by lexical analysis. In the literature, there seems to be agreement on the link between detail and truthfulness [22,24,58]. Furthermore, this trend can be observed in different cultures, such as British, Arabic, or Chinese [59]. This may be due to a lack of ability to provide details when lying [8] or a fear that these details will be checked [60]. In fact, the verifiability approach [61] states that false testimonies tend to provide less verifiable details, but more unverifiable details, such as common knowledge or general details [14]. However, it is suggested that this difference disappears if time is allowed to elapse between coding and interview, due to memory decline in truth-tellers and stability bias in liars [40].

As regards the motivational criteria of content, a greater tendency was found to admit a lack of memory in truthful testimonies, compared with making spontaneous corrections. However, it is believed that when lying, we are more focused on strategic self-presentation in order to appear credible [14]. This means that we are less likely to self-correct and admit memory lapses or doubt our own testimony [21,22]. This tendency is linked to the higher frequency observed in truthful testimonies of verbs in the present tense. Although the literature has associated the present tense with lying [5], in this study, it was observed that the greatest use of this verb tense was focused on the verbs “to believe”, “to remember”, and “to know”. Specifically, they were used in expressions such as “I believe it was...”, “I can’t remember anymore”, or “I don’t know for sure whether”. Therefore, these expressions can be seen as a reflection of the admission of memory failure [5,9,21,22]. However, some studies have associated lying with references to thinking, memory, and other cognitive processes [49,62]. To resolve this inconsistency, it is important not to confuse the admission of lack of memory with self-handicapping strategies [63]. These strategies consist of justifying why certain information cannot be given. They are used in lying because, as we said, fewer verifiable details are offered and the simple fact of admitting a memory failure without justifying the reason is considered detrimental to credibility [63]. In this way, lying avoids criteria related to positive strategic self-presentation efforts (admitting a lack of memory and spontaneous corrections) and, also, information deviating from the script (superfluous details) [15]. This increases the validity of these criteria for detecting truthfulness.

Finally, in general terms, no differences were found in terms of speech disfluencies. This result is consistent with other studies. There is a popular belief that you can detect lying through non-verbal communication. However, despite a large body of research, there seems to be no scientific evidence for various reasons: some signals have not yet been analysed; the measurements are imprecise; the non-verbal expression of lying is related to individual or contextual differences; there is a group of signals with greater discriminative power; there is an imitation of the interviewer’s behaviour; liars and truth-tellers use the same non-verbal strategies [40]. It is important to focus on this last reason, as in this study, we observed a similar pattern between the two conditions, with hesitation and repetition being the most frequent. It is proposed that speech disfluencies are fillers that are linked to the speaker’s *Feeling of Knowing* (FOK), that is with the confidence expressed by the speaker [64]. Similarly, there appears to be the same relationship with *Feeling of Another’s Knowing* (FOAK) or the listener’s perceived confidence [65]. As proposed by the research group of Dinkar and colleagues [66], fillers have various functions that relate in different ways to trust. In this connection, it might be interesting to study spoken language processing in court hearings, relating the different functions of fillers to FOAK.

In the study, ecological validity was sought through intentional non-coding, the distractor counting task, and verbal testimony. However, some authors agree that discrimination ability is lower in the laboratory than in reality [7]. One should bear in mind that we were dealing with a simulated deception setup. For this reason, biases related to the ecological validity of results might occur. Future work should try to examine ecological validity from a simulation environment. In contrast, the advantage of these experiments lies in the control of variables, increasing the validity of the conclusions [5]. For this purpose, the sample was balanced in terms of the gender and age of the participants. However, these variables, along with many others, such as IQ or narrative skills, influence the ability to remember and also to fabricate false testimony [14,67,68,69]. It would be interesting, as future research on deception, to study the linguistic and content characteristics that vary or that remain stable in relation to these variables. Other limitations are related to the selected task from the literature. Systematic replications might benefit from a more balanced set of stimuli regarding language use in testimonies.

In summary, these findings are relevant in the forensics context. Considering barriers, as well as the increasing need to implement current techniques, psycho-linguistics might offer a cutting-edge approach in the field. In particular, professionals are advised to take into account scientific findings in the face of the myths associated with lying. It is also recommended to pay attention to the influence of stereotypes on the testimony. Moreover, this study contributes to the development of NLP tools for deception detection in Spanish. Indeed, Spanish, along with other Romance languages, provides a less-explored scenario for detecting deception and an interesting test bed to evaluate the cross-linguistic validity of earlier studies, which were mainly conducted on English. Nevertheless, the setup of this study is potentially language-independent thanks to the multilingual syntactic representation formalism provided by UD, which guarantees the comparative encoding of language phenomena across different languages [35] and upon which we based the acquisition of the stylometric properties from testimonies. Furthermore, also content and speech disfluencies properties are general enough to be multilingual, as testified by their past use on a corpus of Italian texts [41]. For future research, it is recommended to further explore deception detection in a real context and to take advantage of the synergy between computational linguistics and psychology to increase the sensitivity of the instruments.

## Figures and Tables

**Figure A1:**
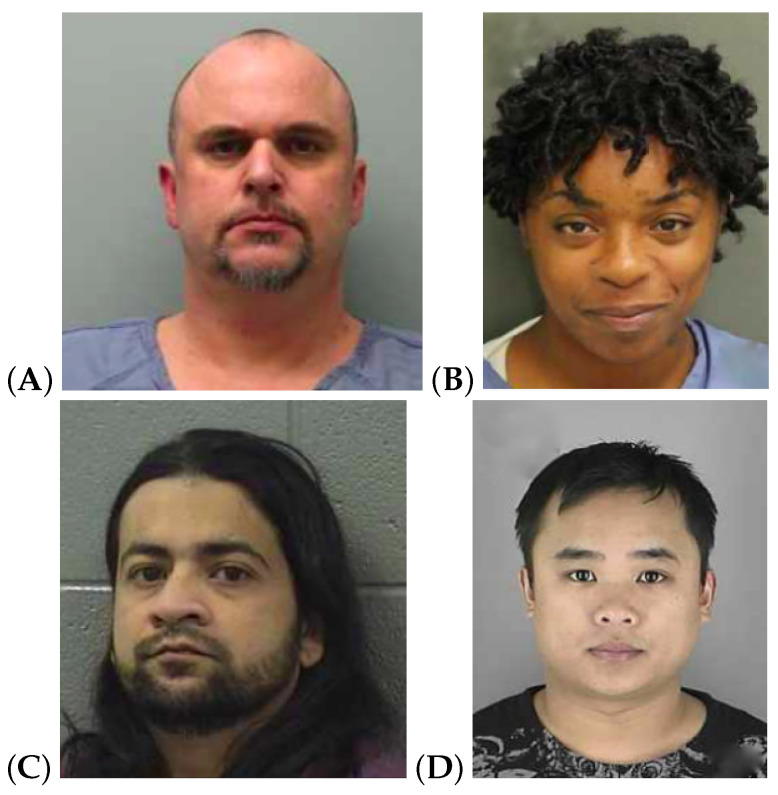
Pictures of characters in the story shown to the study participants: (**A**) is the purse-snatcher, (**B**) is the victim and (**C**) and (**D**) are the pursuers.

**Figure A2:**
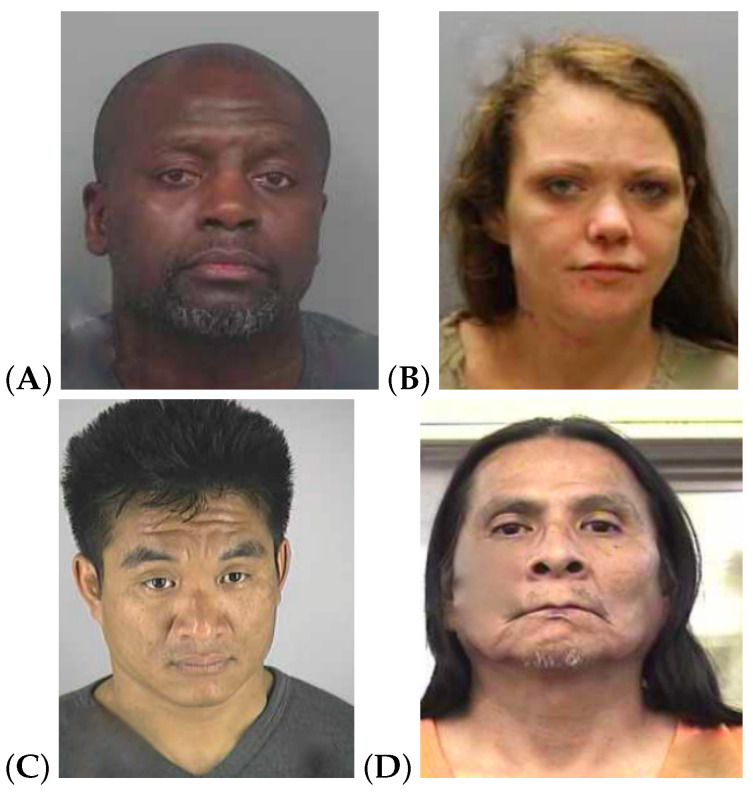
Pictures of characters in the story shown to the study participants: (**A**) is the purse-snatcher, (**B**) is the victim and (**C**) and (**D**) are the pursuers.

**Table 1:** Linguistic indicators aggregated by group.

Linguistic Feature	Label
**Raw Text properties (** ***RawText*** **)**	
Average document length in tokens	n_tokens
Average sentence length	sent_length
Average word length	char_per_tok
**Morphosyntactic information (** ***POSs*** **)**	
Distribution of UD POSs	upos_dist_*
**Inflectional morphology (** ***VerbInflection*** **)**	
Inflectional morphology of lexical verbs and auxiliaries	verbs_*, aux_*
**Verbal Predicate Structure (** ***VerbPredicate*** **)**	
Distribution of verbal heads and verbal roots	verbal_head_dist, verbal_root_perc
Verb arity and distribution of verbs by arity	avg_verb_edges, verbal_arity_*
**Global and local parsed Tree Structures (** ***TreeStructure*** **)**	
Average depth of the whole syntactic tree	tree_depth
Average length of dependency links and of the longest link	avg_links_len, max_links_len
Average length of prepositional chains and distribution by depth	avg_prep_chain_len, prep_dist_1
Average clause length	avg_token_per_clause
**Order of elements (** ***Order*** **)**	
Relative order of subject and object	subj_pre, subj_post, obj_post
**Syntactic relations (** ***SyntacticDep*** **)**	
Distribution of dependency relations	dep_dist_*
**Use of Subordination (** ***Subord*** **)**	
Distribution of subordinate clauses	subordinate_prop_dist
Average length of subordination chains and distribution by depth	avg_subord_chain_len, subordinate_dist_1
Relative order of subordinate clauses	subordinate_post

**Table 2:** Description and examples of the use of content and speech disfluency properties.

Type	Tag	Description	Example
*Content*	**Cognitive criteria**		
	*Contextual information*	Details of time and space in the testimony.	It was <ci>June</ci> and we were in <ci>Valencia</ci>.
	*Superfluous details*	Peripheral and unnecessary details for understanding the facts.	<sd>The ice cream was chocolate and vanilla with macadamia nuts.</sd>
	*Quantity of details*	Descriptions about the place, people, objects, temporal context, etc. Attribute “n” indicates the amount of details provided.	I was walking with <qd n=”2”>my friend Maria to an art exhibition</qd>.
	**Motivational criteria**		
	*Admitting lack of memory*	Raising doubts or acknowledging not remembering a detail.	<lm>I don’t remember the street.</lm>
	*Spontaneous corrections*	Spontaneous self-correction of a detail. Attribute “corrsp” indicates the actual correct word.	He stole her <err corrsp =”handbag”>wallet</err>.
*Speech Disfluencies*	*False start*	Truncated sentences restarted with a new train of thought.	<fs>Then, all of a sudden</fs>.... We were talking and all of a sudden we saw that behind the ice cream stand [...]
	*Repetition*	Words or phrases repeated. Attribute “n” indicates the number of repetitions.	I go with a friend to <rep n=”3”>the</rep> garden of Viveros.
	*Grammatical Corrections*	Self-correction of grammar-related errors. Attribute “corrgr” indicates the actual correct word.	It was <err corrgr="the">a</err> hottest day of the year.
	*Hesitation*	Speech fillers. Attribute “in_text” indicates what should be in the text in place of the filler.	The woman <vac in_text=”” >ehhh</vac> had dark hair

**Table 3:** Frequency distribution as count and relative frequency over the corresponding corpus (%) of lemmas based on their grammatical category in the Truth (T), Simulated Deception (SD) sub-corpora and in the full corpus of testimonies (All). The lexical overlap refers to the rate of unique lemmas shared by T and SD testimonies. Spearman correlations, computed for each POS between the frequency rankings of words shared by T and SD, are all significant (*p* < 0.5).

		Frequency Distribution				
Part-of-Speech		T (%)	SD (%)	All (%)	Lexical Overlap	Spearman Corr.
*Closed-class words*		7685 (54.84%)	5993 (54.01%)	13,678 (54.47%)	64.36%	0.871
	Adpositions	1985 (14.16%)	1473 (13.28%)	3458 (13.77%)	75.00%	0.965
	Auxiliaries	661 (4.72%)	538 (4.85%)	1199 (4.77%)	58.33%	0.964
	Conjunctions	1480 (10.56%)	1260 (11.36%)	2740 (10.91%)	100%	0.952
	Determiners	2060 (14.70%)	1592 (14.35%)	3652 (14.54%)	60.00%	0.965
	Numerals	251 (1.79%)	211 (1.90%)	462 (1.84%)	27.78%	0.900
	Pronouns	1248 (8.91%)	919 (8.28%)	2167 (8.63%)	76.92%	0.867
*Open-class words*		6315 (45.06%)	5089 (45.86%)	11,404 (45.42%)	46.60%	0.748
	Adjectives	425 (3.03%)	513 (4.62%)	938 (3.74%)	32.75%	0.667
	Adverbs	727 (5.19%)	557 (5.02%)	1284 (5.11%)	60.92%	0.797
	Nouns	2634 (18.8%)	2113 (19.04%)	4747 (18.9%)	42.52%	0.803
	Proper nouns	299 (2.13%)	188 (1.69%)	487 (1.94%)	40.00%	0.972
	Verbs	2230 (15.91%)	1718 (15.48%)	3948 (15.72%)	44.69%	0.857
Other		14 (0.1%)	14 (0.13%)	28 (0.11%)	20.00%	-
Total		14,014 (100%)	11,096 (100%)	25,110 (100%)	48.98%	0.846

**Table 4:** Top 10 words by parts-of-speech in Truth (T) and Simulated Deception (SD) testimonies.

	Adjectives		Adverbs		Nouns		Verbs	
	T	SD	T	SD	T	SD	T	SD
1	negro	blanco	no	no	bolso	bolso	ver	ver
2	caluroso	largo	detrás	detrás	hombre	hombre	correr	correr
3	asiático	bueno	más	mucho	mujer	mujer	ir	robar
4	largo	moreno	mucho	entonces	helado	chico	robar	ir
5	blanco	negro	entonces	más	chico	helado	coger	salir
6	bueno	joven	antes	así	amigo	amigo	salir	coger
7	contrario	caluroso	así	también	persona	pelo	ser	ser
8	corto	asiático	también	antes	señor	persona	pasear	pasear
9	moreno	fuerte	ya	menos	ladrón	señor	conseguir	estar
10	fresco	rubio	después	después	día	ladrón	estar	conseguir

**Table 5:** Mean features values in Truth (T) and Simulated Deception (SD) testimonies and their standard deviations (stdev). The “Variation” block reports the percentage of testimonies where the feature value was higher (T > SD), equal (T = SD), or lower (T < SD) in truth than in simulated deception testimonies.

		Mean and Standard Deviation				Variation		
Group	Feature	T	Stdev	SD	Stdev	T > SD	T = SD	T < SD
*Raw Text*	n_tokens	82.19	45.23	64.80	39.75	75.00%	1.04%	23.96%
	n_sentences	4.23	2.10	3.30	1.67	56.25%	28.13%	15.63%
*POS*	upos_dist_ADP	12.64	2.99	12.03	3.44	59.38%	0.52%	40.10%
	upos_dist_VERB	14.74	3.17	14.05	3.54	58.85%	0.52%	40.63%
	upos_dist_PRON	7.84	2.86	7.22	2.97	57.81%	0.52%	41.67%
	upos_dist_ADV	4.38	2.37	3.90	2.68	55.21%	2.08%	42.71%
	upos_dist_PROPN	1.92	1.62	1.35	1.50	51.56%	22.92%	25.52%
	upos_dist_NOUN	16.96	2.80	17.90	3.23	39.58%	1.56%	58.85%
	upos_dist_CCONJ	5.60	2.47	6.44	2.79	35.94%	2.08%	61.98%
	upos_dist_ADJ	2.13	2.06	4.07	2.84	23.96%	6.25%	69.79%
	upos_dist_INTJ	0.01	0.10	0.06	0.37	1.04%	94.79%	4.17%
*VerbInflection*	verbs_form_dist_Inf	15.15	10.70	13.18	11.36	48.44%	13.54%	38.02%
	verbs_tense_dist_Pres	19.35	24.89	15.67	23.80	39.58%	36.46%	23.96%
	aux_tense_dist_Pres	14.08	28.83	9.87	23.66	19.27%	71.35%	9.38%
	aux_mood_dist_Ind	91.07	27.09	84.83	35.31	14.06%	77.08%	8.85%
*TreeStructure*	max_links_len	15.11	7.71	13.24	7.18	58.33%	4.69%	36.98%
	n_prepositional_chains	2.59	2.45	1.94	1.84	45.31%	25.52%	29.17%
	avg_token_per_clause	6.71	1.40	7.01	1.73	41.67%	0.52%	57.81%
*SyntacticDep*	dep_dist_obl	6.22	2.60	5.41	2.71	57.81%	0.52%	41.67%
	dep_dist_acl:relcl	2.32	2.03	1.84	1.85	55.21%	8.33%	36.46%
	dep_dist_iobj	3.35	2.04	3.76	2.61	43.75%	2.08%	54.17%
	dep_dist_fixed	1.01	1.27	0.69	1.12	39.58%	32.29%	28.13%
	dep_dist_cc	5.51	2.43	6.32	2.78	35.94%	2.08%	61.98%
	dep_dist_conj	4.16	2.39	5.33	3.11	34.90%	2.60%	62.50%
	dep_dist_amod	1.60	1.62	2.93	2.22	22.92%	9.90%	67.19%
	dep_dist_nsubj:pass	0.08	0.37	0.02	0.14	4.69%	93.23%	2.08%
*Subord*	subordinate_proposition_dist	57.87	15.03	53.70	16.84	53.13%	8.85%	38.02%
	avg_subordinate_chain_len	1.22	0.27	1.16	0.36	41.67%	31.77%	26.56%
	principal_proposition_dist	42.13	15.03	46.30	16.84	38.02%	8.85%	53.13%
	subordinate_dist_2	17.05	19.71	13.96	22.15	36.98%	37.50%	25.52%

**Table 6:** Distribution of tags in Truth (T) and Simulated Deception (SD) testimonies and variation analysis.

		Frequency		Variation		
Type	TAG	T	SD	T > SD	T = SD	T < SD
*Cognitive criteria*	Contextual information	288	180	65.63	27.08	7.29
	Superfluous details	190	132	52.08	39.58	8.34
	Quantity of details	478	312	80.21	14.58	5.21
	Type average	956	624	65.97	27.08	6.95
*Motivational criteria*	Lack of memory	65	19	21.87	75.00	3.13
	Spontaneous corrections	16	8	7.29	89.06	3.65
	Type average	81	27	14.58	82.03	3.39
*Speech* *disfluencies*	False start	79	56	21.35	67.19	11.46
	Repetition	159	158	25.52	47.40	27.08
	Grammatical corrections	42	37	15.63	70.83	13.54
	Hesitation	557	567	33.85	22.92	43.23
	Type average	837	818	24.09	52.08	23.83

## Data Availability

Not applicable.

## Sample Availability

The database used for this study can be obtained upon request to the corresponding author.

## Abbreviations

The following abbreviations are used in this manuscript:

NLP	Natural Language Processing
T	Truthful testimonies
SD	Simulation Deceptive testimonies
CBCA	Criteria-Based Content Analysis
RM	Reality Monitoring
LIWC	Linguistic Inquiry and Word Count
POS	Part-Of-Speech

## Appendix A. Stories Presented to Participants

We report the stories describing the robbery presented to participants in the study. These stories were adapted from those reported in [25], which in turn were based on Helm’s investigation [26]. The same images were used as in the study cited above, which, according to the authors [25], were acquired from the public online repository bustedmugshots.com accessed on 6 February 2023.

### Appendix A.1. Story A

***Original version in Spanish:*** El miércoles pasado, un sorprendente incidente de robo de cartera ocurrió fuera de una tienda de ropa, en calle Colón. Tu amiga María y tú, habíais caminado por el centro de Valencia para llegar a la gran inauguración de una exposición de arte. María acababa de terminar de contarte su día cuando visteis una conmoción delante vuestra, así que os parasteis a ver qué pasaba. Viste a una persona (Figure A1A) acercarse y golpear violentamente a una mujer (Figure A1B) contra el pavimento. La mujer intentó agarrarse con fuerza a su bolso, mientras el hombre la arrastraba durante bastante tiempo, hasta que pudo arrancarle el bolso completamente. A continuación, el hombre corrió rápidamente en dirección contraria a la tuya, hacia Alameda. Viste a dos personas (Figure A1C,D) intentar seguir al ladrón de bolsos, pero no lo encontraron.

***English translation:*** Last Wednesday, a surprising incident of purse snatching occurred outside a clothing shop in Calle Colón. You and your friend, Maria, had been walking through the centre of Valencia to get to the grand opening of an art exhibition. Maria had just finished telling you about her day when you saw a commotion in front of you, so you stopped to see what was going on. You saw a person (Figure A1A) approaching and violently hitting a woman (Figure A1B) to the ground. The woman tried to hold on tightly to her handbag, while the man dragged her for quite some time until he was able to tear her handbag completely out of her hands. The man then quickly ran in the opposite direction to you, towards Alameda. You saw two people (Figure A1C,D) trying to follow the purse snatcher, but they did not find him.

### Appendix A.2. Story B

***Original version in Spanish:*** Habías caminado por la acera de los Jardines de Viveros en Valencia con un amigo. Era un caluroso día de junio, pero el viento deslizaba una brisa fresca por tu hombro. La brisa era muy refrescante, ya que la estación meteorológica indicó que era el día más caluroso del año. En ese momento notaste un movimiento delante de ti, detrás del puesto de helados, así que miraste para ver qué pasaba. Viste a una persona (Figure A2A) acercarse rápidamente a otra (Figure A2B) que estaba pidiendo dos helados: uno, de chocolate y, el otro, de vainilla y nueces de Macadamia. El hombre rápidamente le arrebató el bolso de los hombros, cuando ella levantó la mano para coger su pedido. El hombre corrió rápidamente hacia el aparcamiento con el bolso de la mujer. También viste a dos personas (Figure A2C,D) que intentaron seguir al ladrón de bolsos, pero que finalmente no pudieron alcanzarlo.

***English translation:*** You had walked along the pavement of the Jardines de Viveros in Valencia with a friend. It was a hot day in June, but the wind was blowing a cool breeze across your shoulder. The breeze was very refreshing, as the weather station indicated that it was the hottest day of the year. At that moment you noticed a movement in front of you, behind the ice cream stand, so you looked to see what was going on. You saw a person (Figure A2A) quickly approach another person (Figure A2B) who was ordering two ice creams: one of chocolate and the other one of vanilla and macadamia nut. The man quickly snatched the bag from her shoulders as she raised her hand to take her order. The man quickly ran into the car park with the woman’s handbag. You also saw two people (Figure A2C,D) who tried to follow the purse snatcher, but were unable to catch up with him in the end.

## Appendix B. Linguistic Features

### Appendix B.1. Wilcoxon Signed-Rank Test Results

**Table A1:** For each linguistic feature, we report the W score of the Wilcoxon signed-rank test (‘*W*’) computed for the feature values obtained on retellings of T and SD, the *p*-value (‘*p*’), and the effect size score (“*r*”). Features that show a significant variation (*p* < 0.05) between the two corpora are marked as “(*)”; non-significantly varying features are marked as “(-)”.

		Wilcoxon		
Group	Feature	W	*p*	*r*
*Raw Text*	n_sentences (* )	1662.5	0.000	0.34
	n_tokens (*)	3091.5	0.000	0.31
	tokens_per_sent (- )	8850.5	0.866	0.00
	char_per_tok (-)	7726	0.059	0.14
*POS*	upos_dist_ADJ (*)	2588.5	0.000	0.50
	upos_dist_ADP (*)	7344.5	0.017	0.13
	upos_dist_ADV (*)	7397	0.047	0.14
	upos_dist_AUX (-)	8330.5	0.536	0.03
	upos_dist_CCONJ (*)	5835.5	0.000	0.22
	upos_dist_DET (-)	8477.5	0.433	0.02
	upos_dist_INTJ (*)	8	0.047	0.14
	upos_dist_NOUN (*)	6444	0.001	0.22
	upos_dist_NUM (-)	8040.5	0.373	0.01
	upos_dist_PRON (*)	7139	0.008	0.16
	upos_dist_PROPN (*)	3112	0.000	0.24
	upos_dist_SCONJ (-)	7300.5	0.149	0.07
	upos_dist_VERB (*)	6910.5	0.003	0.15
	upos_dist_X (-)	28.5	0.132	0.11
*Verb Inflection*	verbs_tense_dist_Fut (-)	23.5	0.398	0.07
	verbs_tense_dist_Imp (-)	5949.5	0.335	0.06
	verbs_tense_dist_Past (-)	6180	0.226	0.06
	verbs_tense_dist_Pres (*)	2783.5	0.013	0.16
	verbs_mood_dist_Cnd (-)	3	0.225	0.00
	verbs_mood_dist_Ind (-)	331	0.045	0.03
	verbs_mood_dist_Sub (-)	9	0.207	0.11
	verbs_form_dist_Fin (-)	727	0.076	0.07
	verbs_form_dist_Ger (-)	4.5	0.207	0.08
	verbs_form_dist_Inf (*)	66.5	0.635	0.12
	verbs_form_dist_Part (-)	48	0.177	0.02
	verbs_num_pers_dist_+ (-)	95	0.190	0.13
	verbs_num_pers_dist_Plur+1 (-)	2303	0.445	0.00
	verbs_num_pers_dist_Plur+3 (-)	5846	0.206	0.09
	verbs_num_pers_dist_Sing+ (-)	120	0.583	0.10
	verbs_num_pers_dist_Sing+1 (-)	1867	0.468	0.04
	verbs_num_pers_dist_Sing+3 (-)	6162.5	0.174	0.07
	aux_tense_dist_Imp (-)	3292.5	0.303	0.07
	aux_tense_dist_Past (-)	1887	0.767	0.04
	aux_tense_dist_Pres (*)	476.5	0.014	0.10
	aux_mood_dist_Cnd (-)	513	0.441	0.03
	aux_mood_dist_Ind (*)	1178.5	0.708	0.13
	aux_mood_dist_Sub (-)	59.5	0.051	0.02
	aux_form_dist_Fin (-)	7981.5	0.276	0.08
	aux_form_dist_Ger (-)	6987	0.414	0.08
	aux_form_dist_Inf (-)	5613.5	0.034	0.02
	aux_form_dist_Part (-)	2536	0.092	0.07
	aux_num_pers_dist_Plur+1 (-)	366.5	0.558	0.09
	aux_num_pers_dist_Plur+3 (-)	2913	0.465	0.07
	aux_num_pers_dist_Sing+1 (-)	59.5	0.977	0.00
	aux_num_pers_dist_Sing+3 (-)	3651	0.070	0.10
*Verb Predicate*	verbal_head_per_sent (-)	7036	0.177	0.04
	verbal_root_perc (-)	1319.5	0.149	0.13
	avg_verb_edges (-)	7524	0.088	0.11
	verb_edges_dist_0 (-)	1571.5	0.816	0.03
	verb_edges_dist_1 (-)	7200.5	0.536	0.06
	verb_edges_dist_2 (-)	7748	0.129	0.10
	verb_edges_dist_3 (-)	8211.5	0.973	0.01
	verb_edges_dist_4 (-)	7410.5	0.761	0.04
	verb_edges_dist_5 (-)	2972	0.577	0.01
	verb_edges_dist_6 (-)	628	0.927	0.01
*Tree Structure*	avg_max_depth (-)	8069.5	0.814	0.03
	avg_token_per_clause (*)	7397	0.021	0.10
	avg_max_links_len (-)	8238	0.326	0.06
	avg_links_len (-)	8027	0.136	0.07
	max_links_len (*)	6211	0.002	0.18
	avg_prepositional_chain_len (-)	2060.5	0.251	0.08
	n_prepositional_chains (*)	3203.5	0.000	0.16
	prep_dist_1 (-)	2287	0.745	0.02
	prep_dist_2 (-)	551.5	0.146	0.10
	prep_dist_3 (-)	11	0.172	0.08
*Order*	obj_pre (-)	6950.5	0.112	0.09
	obj_post (-)	7116.5	0.176	0.07
	subj_pre (-)	2473	0.728	0.02
	subj_post (-)	1732	0.112	0.11
*SyntacticDep*	dep_dist_acl (-)	713.5	0.636	0.01
	dep_dist_acl:relcl (*)	5991	0.008	0.15
	dep_dist_advcl (-)	6645.5	0.055	0.14
	dep_dist_advmod (-)	7698.5	0.141	0.09
	dep_dist_amod (*)	2734.5	0.000	0.46
	dep_dist_appos (-)	1825.5	0.125	0.07
	dep_dist_aux (-)	6765	0.081	0.11
	dep_dist_aux:pass (-)	283.5	0.207	0.14
	dep_dist_case (-)	8570	0.368	0.06
	dep_dist_cc (*)	5899	0.000	0.21
	dep_dist_ccomp (-)	1686.5	0.259	0.13
	dep_dist_conj (*)	5463	0.000	0.28
	dep_dist_cop (-)	3100.5	0.127	0.13
	dep_dist_csubj (-)	102	0.426	0.03
	dep_dist_dep (-)	2	0.593	0.04
	dep_dist_det (-)	8819	0.648	0.00
	dep_dist_fixed (*)	3034.5	0.004	0.21
	dep_dist_flat (-)	2	0.593	0.07
	dep_dist_iobj (*)	7342.5	0.039	0.11
	dep_dist_mark (-)	7308	0.059	0.12
	dep_dist_nmod (-)	7833.5	0.950	0.01
	dep_dist_nsubj (-)	7731.5	0.098	0.12
	dep_dist_nsubj:pass (*)	13	0.023	0.12
	dep_dist_nummod (-)	8272.5	0.565	0.08
	dep_dist_obj (-)	8052	0.219	0.05
	dep_dist_obl (*)	6978.5	0.004	0.22
	dep_dist_parataxis (-)	2660	0.386	0.04
	dep_dist_root (-)	8689	0.702	0.02
	dep_dist_xcomp (-)	4239.5	0.441	0.00
*Subord*	principal_proposition_dist (*)	5733	0.003	0.17
	subordinate_proposition_dist (*)	5723.5	0.003	0.17
	subordinate_post (-)	1818.5	0.823	0.07
	subordinate_pre (-)	1468.5	0.115	0.11
	avg_subordinate_chain_len (*)	3157.5	0.007	0.15
	subordinate_dist_1 (-)	3761	0.196	0.07
	subordinate_dist_2 (*)	2742.5	0.020	0.17
	subordinate_dist_3 (-)	243.5	0.701	0.06
	subordinate_dist_4 (-)	3.5	0.581	0.07

### Appendix B.2. Feature Values and Variation Analysis

**Table A2:** Linguistic features acquired from transcriptions, organised by group: for each feature, we report their mean value and standard deviation in the two sub-corpora of Truthful (T) and Simulated Deception (SD) testimonies. In the “Variation” block, we report the percentage of testimonies for which the feature value is higher (T > SD), equal (T = SD), or lower (T < SD) in T or SD. Features that show a significant variation (*p* < 0.05) between the two corpora according to the Wilcoxon signed-rank test (see Table A1) are marked as “(*)”; non-significantly varying features are marked as “(-)”.

		Mean & Standard Deviation				Variation (%)		
Group	Feature	T	Stdev	SD	Stdev	TSD	T = SD	TSD
*Raw Text*	n_sentences (*)	4.23	2.10	3.30	1.67	56.25	28.13	15.63
	n_tokens (*)	82.19	45.23	64.80	39.75	75.00	1.04	23.96
	tokens_per_sent (-)	19.88	5.93	19.91	6.31	50.00	1.56	48.44
	char_per_tok (-)	4.19	0.24	4.14	0.28	51.56	0.52	47.92
*POS*	upos_dist_ADJ (*)	2.13	2.06	4.07	2.84	23.95	6.25	69.79
	upos_dist_ADP (*)	12.64	2.99	12.03	3.44	59.37	0.52	40.10
	upos_dist_ADV (*)	4.38	2.37	3.90	2.68	55.21	2.08	42.71
	upos_dist_AUX (-)	4.20	2.49	4.04	2.68	50.52	2.60	46.88
	upos_dist_CCONJ (*)	5.60	2.47	6.44	2.79	35.94	2.08	61.98
	upos_dist_DET (-)	13.36	2.58	13.50	2.76	45.31	1.04	53.65
	upos_dist_INTJ (*)	0.01	0.10	0.06	0.37	1.04	94.79	4.17
	upos_dist_NOUN (*)	16.96	2.80	17.90	3.23	39.58	1.56	58.85
	upos_dist_NUM (-)	1.90	1.08	1.97	1.59	44.27	3.13	52.60
	upos_dist_PRON (*)	7.84	2.86	7.22	2.97	57.81	0.52	41.67
	upos_dist_PROPN (*)	1.92	1.62	1.35	1.50	51.56	22.92	25.52
	upos_dist_SCONJ (-)	3.70	2.44	3.42	2.42	52.08	5.21	42.71
	upos_dist_VERB (*)	14.74	3.17	14.05	3.54	58.85	0.52	40.63
	upos_dist_X (-)	0.07	0.31	0.03	0.18	5.73	92.71	1.56
*Verb Inflection*	verbs_tense_dist_Fut (-)	0.51	3.16	0.23	1.67	3.65	94.27	2.08
	verbs_tense_dist_Imp (-)	25.14	18.38	26.78	21.31	37.50	16.15	46.35
	verbs_tense_dist_Past (-)	55.01	26.00	57.31	26.56	39.58	13.54	46.88
	verbs_tense_dist_Pres (*)	19.35	24.89	15.67	23.80	39.58	36.46	23.96
	verbs_mood_dist_Cnd (-)	2.59	7.92	3.61	11.88	11.46	75.00	13.54
	verbs_mood_dist_Ind (-)	95.59	11.37	95.42	13.85	17.71	63.54	18.75
	verbs_mood_dist_Sub (-)	1.22	4.85	0.45	2.48	7.29	89.06	3.65
	verbs_form_dist_Fin (-)	56.92	17.75	58.66	18.10	45.83	2.60	51.56
	verbs_form_dist_Ger (-)	18.52	10.76	17.47	13.27	44.79	9.90	45.31
	verbs_form_dist_Inf (*)	15.15	10.70	13.18	11.36	48.44	13.54	38.02
	verbs_form_dist_Part (-)	9.41	13.46	10.69	14.20	25.00	42.19	32.81
	verbs_num_pers_dist_+ (-)	1.51	5.63	0.84	4.90	8.33	88.02	3.65
	verbs_num_pers_dist_Plur+1 (-)	13.55	19.68	13.98	19.89	23.44	47.92	28.65
	verbs_num_pers_dist_Plur+3 (-)	24.08	20.48	23.55	26.25	45.83	15.63	38.54
	verbs_num_pers_dist_Sing+ (-)	1.04	4.13	0.77	4.47	7.81	88.02	4.17
	verbs_num_pers_dist_Sing+1 (-)	8.47	13.55	7.99	15.70	26.04	53.13	20.83
	verbs_num_pers_dist_Sing+3 (-)	50.75	22.16	52.86	27.07	39.58	13.02	47.40
	aux_tense_dist_Imp (-)	62.58	38.07	58.70	40.65	33.85	36.98	29.17
	aux_tense_dist_Past (-)	15.53	26.39	16.85	29.45	22.92	54.17	22.92
	aux_tense_dist_Pres (*)	14.08	28.83	9.87	23.66	19.27	71.35	9.38
	aux_mood_dist_Cnd (-)	0.39	3.22	0.14	1.37	1.56	97.40	1.04
	aux_mood_dist_Ind (*)	91.07	27.09	84.83	35.31	14.06	77.08	8.85
	aux_mood_dist_Sub (-)	0.72	4.17	0.45	3.14	2.60	95.83	1.56
	aux_form_dist_Fin (-)	89.97	27.15	83.83	35.29	19.27	67.71	13.02
	aux_form_dist_Ger (-)	0.92	7.71	0.19	1.87	2.08	96.88	1.04
	aux_form_dist_Inf (-)	0.76	3.66	1.29	8.02	4.17	91.15	4.69
	aux_form_dist_Part (-)	1.06	5.19	0.62	4.21	5.21	91.15	3.65
	aux_num_pers_dist_Plur+1 (-)	4.46	12.74	4.55	17.55	12.50	79.17	8.33
	aux_num_pers_dist_Plur+3 (-)	17.63	26.50	16.64	28.47	31.77	41.67	26.56
	aux_num_pers_dist_Sing+1 (-)	1.45	6.95	1.28	6.63	3.13	92.19	4.69
	aux_num_pers_dist_Sing+3 (-)	68.59	34.30	62.80	39.63	38.54	30.73	30.73
*Verb Predicate*	verbal_head_per_sent (-)	3.07	1.03	2.98	1.12	52.60	7.29	40.10
	verbal_root_perc (-)	91.84	14.25	93.61	13.47	17.71	58.33	23.96
	avg_verb_edges (-)	2.61	0.37	2.68	0.42	44.79	2.60	52.60
	verb_edges_dist_0 (-)	2.65	4.54	3.09	6.46	21.88	58.33	19.79
	verb_edges_dist_1 (-)	14.45	11.37	13.78	12.38	47.40	9.38	43.23
	verb_edges_dist_2 (-)	29.96	14.42	27.64	16.63	52.08	2.08	45.83
	verb_edges_dist_3 (-)	30.54	14.43	31.08	18.03	46.88	5.73	47.40
	verb_edges_dist_4 (-)	16.65	12.57	17.91	15.79	42.71	9.38	47.92
	verb_edges_dist_5 (-)	4.44	6.44	5.11	8.62	30.21	41.67	28.13
	verb_edges_dist_6 (-)	1.31	3.20	1.39	4.06	13.02	73.96	13.02
*Tree Structure*	avg_max_depth (-)	4.36	1.11	4.40	1.23	50.52	5.73	43.75
	avg_token_per_clause (*)	6.71	1.40	7.01	1.73	41.67	0.52	57.81
	avg_max_links_len (-)	9.23	3.71	8.93	4.09	54.17	1.56	44.27
	avg_links_len (-)	2.49	0.35	2.45	0.40	55.73	0.52	43.75
	max_links_len (*)	15.11	7.71	13.24	7.18	58.33	4.69	36.98
	avg_prepositional_chain_len (-)	0.93	0.47	0.88	0.46	28.65	49.48	21.88
	n_prepositional_chains (*)	2.59	2.45	1.94	1.84	45.31	25.52	29.17
	prep_dist_1 (-)	73.72	39.07	74.69	39.94	23.44	49.48	27.08
	prep_dist_2 (-)	8.32	19.88	6.05	17.30	17.71	72.40	9.90
	prep_dist_3 (-)	0.77	3.61	0.51	3.41	2.60	95.31	2.08
*Order*	obj_pre (-)	34.72	18.64	31.83	18.86	52.60	6.77	40.63
	obj_post (-)	65.28	18.64	67.65	19.35	41.15	6.77	52.08
	subj_pre (-)	82.32	28.64	82.74	29.98	23.44	47.40	29.17
	subj_post (-)	14.03	23.88	9.97	19.11	28.65	52.08	19.27
*SyntacticDep*	dep_dist_acl (-)	0.26	0.65	0.30	0.73	13.54	71.35	15.10
	dep_dist_acl:relcl (*)	2.32	2.03	1.84	1.85	55.21	8.33	36.46
	dep_dist_advcl (-)	3.14	1.97	2.85	2.19	53.13	7.29	39.58
	dep_dist_advmod (-)	3.76	2.19	3.46	2.43	51.56	2.60	45.83
	dep_dist_amod (*)	1.60	1.62	2.93	2.22	22.92	9.90	67.19
	dep_dist_appos (-)	0.78	1.07	0.68	1.11	28.65	51.04	20.31
	dep_dist_aux (-)	3.12	2.34	2.74	2.24	50.52	7.29	42.19
	dep_dist_aux:pass (-)	0.19	0.52	0.12	0.47	11.98	80.21	7.81
	dep_dist_case (-)	11.40	3.02	11.21	3.42	53.13	0.00	46.88
	dep_dist_cc (*)	5.51	2.43	6.32	2.78	35.94	2.08	61.98
	dep_dist_ccomp (-)	0.50	0.82	0.41	0.85	26.56	54.17	19.27
	dep_dist_conj (*)	4.16	2.39	5.33	3.11	34.90	2.60	62.50
	dep_dist_cop (-)	0.84	1.12	1.04	1.35	31.25	36.98	31.77
	dep_dist_csubj (-)	0.07	0.29	0.09	0.38	5.21	88.54	6.25
	dep_dist_dep (-)	0.01	0.09	0.00	0.06	1.04	98.44	0.52
	dep_dist_det (-)	13.39	2.59	13.45	2.76	45.83	0.52	53.65
	dep_dist_fixed (*)	1.01	1.27	0.69	1.12	39.58	32.29	28.13
	dep_dist_flat (-)	0.02	0.17	0.01	0.09	1.04	98.44	0.52
	dep_dist_iobj (*)	3.35	2.04	3.76	2.61	43.75	2.08	54.17
	dep_dist_mark (-)	4.66	2.73	4.15	2.53	55.73	3.13	41.15
	dep_dist_nmod (-)	3.31	2.36	3.29	2.31	46.88	7.81	45.31
	dep_dist_nsubj (-)	3.96	2.05	4.34	2.50	46.88	1.56	51.56
	dep_dist_nsubj:pass (*)	0.08	0.37	0.02	0.14	4.69	93.23	2.08
	dep_dist_nummod (-)	1.84	1.09	1.77	1.57	47.92	3.13	48.96
	dep_dist_obj (-)	6.77	2.26	6.56	2.69	53.65	1.56	44.79
	dep_dist_obl (*)	6.22	2.60	5.41	2.71	57.81	0.52	41.67
	dep_dist_parataxis (-)	0.62	1.02	0.67	1.04	26.04	43.75	30.21
	dep_dist_root (-)	5.46	1.53	5.49	1.62	48.44	1.56	50.00
	dep_dist_xcomp (-)	1.10	1.38	1.03	1.32	38.54	29.69	31.77
*Subord*	principal_proposition_dist (*)	42.13	15.03	46.30	16.84	38.02	8.85	53.13
	subordinate_proposition_dist (*)	57.87	15.03	53.70	16.84	53.13	8.85	38.02
	subordinate_post (-)	90.15	20.70	89.96	23.97	20.83	55.21	23.96
	subordinate_pre (-)	9.33	19.66	6.91	17.71	26.04	55.73	18.23
	avg_subordinate_chain_len (*)	1.22	0.27	1.16	0.36	41.67	31.77	26.56
	subordinate_dist_1 (-)	79.84	21.64	80.37	27.79	31.25	31.77	36.98
	subordinate_dist_2 (*)	17.05	19.71	13.96	22.15	36.98	37.50	25.52
	subordinate_dist_3 (-)	2.42	7.76	2.45	10.28	9.90	83.33	6.77
	subordinate_dist_4 (-)	0.17	1.47	0.09	1.20	1.56	97.92	0.52
